# Farming system shapes rhizosphere microbiota and root gene expression in common bean

**DOI:** 10.3389/fpls.2026.1749874

**Published:** 2026-03-26

**Authors:** Marta Suarez-Fernandez, Carmen García-Fernández, Juan José Ferreira, Ana Campa

**Affiliations:** Plant Genetics Group, Regional Service for Agrofood Research and Development (SERIDA), Villaviciosa, Spain

**Keywords:** soil microbial diversity, soil microbiome, organic farming, phaseolus vulgaris, RNA-seq

## Abstract

The rhizosphere is a dynamic interface where plant roots and microorganisms interact through the exchange of metabolites and signaling molecules. This study evaluated the impact of organic and conventional farming on the rhizosphere microbiota and root gene expression in common bean by integrating metabarcoding (16S rRNA and ITS) and RNA sequencing (RNA-seq) approaches. Bacterial alpha diversity was higher in the rhizosphere of plants grown under conventional than under the organic system (2961 vs. 1532 Amplicon Sequence Variants (ASVs) observed), whereas fungal alpha diversity was greater in the organic system (372 vs. 321 ASVs observed). The fungi-to-prokaryote ratio was approximately twofold higher in organic systems. Organic farming promoted *Funneliformis*, *Metarhizium*, *Chitinophaga*, and *Rhizobium*, while conventional farming favored *Pirellula*, *Terrimonas*, and *Mortierella*. Transcriptomic analysis identified 5511 differentially expressed genes (DEGs), of which 1085 showed |log_2_FC| ≥ 2, mainly upregulated under organic conditions. These genes were enriched in functions related to secondary metabolism, redox homeostasis, hormone signaling, nodulation, and nutrient transport. DEGs involved in the synthesis of root exudate metabolites, including fatty acids, indolic compounds, and organic acids, were also identified, highlighting their potential role in microbial recruitment. Downregulated genes were associated with cell cycle and kinase activity. Correlation analyses linked beneficial fungal taxa with the induction of genes related to plant growth, defense, and symbiosis. This work provides a basis for future studies aimed at identifying key genes involved in root development and plant–microbe interactions, potentially improving breeding programs for cultivar resilience and efficiency.

## 1 Introduction

Soil constitutes a fundamental resource for agricultural production, providing physical support, water, and essential nutrients to crops (Delgado and Gómez, 2024). Beyond its physicochemical properties -such as texture, pH, conductivity, organic matter, and nutrient composition, soil hosts highly diverse microbial communities that play key roles in nutrient cycling, organic matter decomposition, plant nutrition, and health (Sahu et al., 2017; Vincze et al., 2024). The narrow zone surrounding plant roots, known as the rhizosphere, represents a dynamic interface where plants and microorganisms interact through the exchange of metabolites and signaling molecules. These interactions influence plant growth, stress tolerance, and pathogen response, highlighting the critical role of the soil–microbiome–rhizosphere system in crop yield and sustainable agroecosystems (Huang et al., 2014; Gupta et al., 2021; Dini‐Andreote et al., 2025; Gahlot, 2026).

Plant roots respond to the soil environment spatiotemporally by avoiding stressful soil environments and proliferating in more favourable niches (Jin et al., 2017). Roots also selectively recruit microbes through exudates, improving plant nutrition and productivity (Pantigoso et al., 2022). The composition and biodiversity of the soil microbiota are influenced by management practices and farming systems (Oehl et al., 2004; Hartmann et al., 2015; Maretto et al., 2023; Suarez-Fernandez et al., 2025). Organic farming systems avoid synthetic inputs such as fertilizers or pesticides, and promote ecological processes, biodiversity, and soil health. Moreover, it has gained attention for its potential to enhance soil biodiversity and promote more sustainable crops with low inputs (Lori et al., 2017; Montgomery and Biklé, 2021).

The common bean (*Phaseolus vulgaris* L.) is one of the most important grain legumes worldwide, serving as a major source of protein, dietary fiber, and micronutrients for human consumption (Rodríguez Madrera et al., 2021; Añazco et al., 2023). Common bean yield is strongly influenced by root system architecture, which determines water and nutrient acquisition efficiency as well as responses to biotic and abiotic stresses (Sofi et al., 2021). Thus, increasing yields may be facilitated by a deeper understanding of root–soil interactions. Studies on root phenotypes in *P. vulgaris* have revealed substantial genetic variability in root architecture. Quantitative trait *loci* (QTL) associated with traits such as root dry weight, root length, branching, and basal root number have been mapped (Singh et al., 2019). Many QTL studies focused on root system architecture in response to abiotic stresses such as low phosphorus availability, aluminum toxicity and drought (López-Marín et al., 2009; Asfaw and Blair, 2012; Sofi et al., 2021). A limited number of studies have investigated the rhizosphere microbiome associated with common bean cultivars, highlighting the complex interplay among soil microbial communities, bean genotype, agronomic practices, and stress conditions (Park et al., 2023; López Romo et al., 2025). Together, these findings highlight the relevance of root traits and plant–microbe interactions for crop management and breeding programs.

High-throughput sequencing has provided powerful tools to explore DNA and RNA variation, providing novel perspectives for the study of plant–microbe interactions. Metabarcoding enables the characterization of microbial community composition and diversity, offering insights into the taxonomic structure of root-associated microbial communities (Abdelfattah et al., 2018; Semenov, 2021). On the other hand, whole-transcriptome RNA-sequencing (RNA-seq) allows for the analyses of changes in complete transcript sets and their quantification for a specific developmental stage or physiological conditions (Wang et al., 2009). Combining both methodologies offers an integrative framework to link plant genetic responses with shifts in rhizosphere microbial communities, thereby opening new opportunities to understand the molecular and ecological mechanisms that shape plant–microbe interactions (Kumar et al., 2023).

We hypothesize that organic and conventional farming systems differentially shape rhizosphere microbial communities and root gene expression, reflecting specific plant–microbe interactions. This study aims to characterize the rhizosphere microbiota and identify differentially expressed genes in common bean roots in response to two soil management practices: organic and conventional systems. These analyses could provide insight into bean root *loci* that contribute to adaptation to organic farming and low-input systems.

## 2 Materials and methods

### 2.1 Field trials

This study was conducted in two adjacent open-field soils located in Villaviciosa (Asturias, Spain; 43°28’27.0″ N, 5°26’30.1″ W), separated by approximately 150 m and sharing the same topography and local microclimatic conditions. One field had been managed under a conventional farming (CF) system for the last 21 years, while the other had been managed under organic farming (OF) practices for the last 8 years, as previously described by Suarez-Fernandez et al. (2025). In the organic field, fertilization was carried out using green manure within a bean–ryegrass rotation system (bean in summer, ryegrass in winter), with minimal application of organic pesticides for pest and disease control. In the conventional field, two crop cycles per year were developed using inorganic fertilizers and synthetic pesticides.

Field trials were conducted from 15 May to 15 July 2024. For each soil type, two linear plot of 5 meters in length was planted with 30 bean plants per soil type. Plots were mulched with plastic for weed control. The bean genotype A25, derived from an old cultivar obtained through local landrace selection was used. Line A25 has an indeterminate climbing habit and it is classified as market class Fabada (Ferreira et al., 2012). A single genotype was selected to minimize genetic variability and to specifically assess the effects of long-term soil management on rhizosphere microbiota and root gene expression.

### 2.2 Sampling for metabarcoding and RNA-seq analyses

Roots from line A25 were sampled at early flowering stage, collecting five individual plants per farming system (**Supplementary Figure 1**). Each plant was treated as an independent biological replicate. After carefully removing the soil, the roots were excised, flash-frozen in liquid nitrogen, and stored at −80 °C before RNA extraction. For metabarcoding analysis rhizosphere soil (loosely adhering to the roots) was collected, immediately frozen and stored at -80 °C until processing.

All root and rhizosphere samples were collected on the same day and processed simultaneously to minimize technical variability. Sampling order was randomized across farming systems, and samples were randomized during RNA and DNA extraction as well as during library preparation and sequencing to avoid potential batch effects.

Temperature, relative humidity, and precipitation were monitored throughout 2024, including the whole bean crop cycle (**Supplementary Figure 2**). Additionally, physicochemical analyses of the soil characteristics of the conventional and organic trials were conducted by the company KUDAM (Laboratorio Kudam S.L., Alicante, Spain). Each soil was sampled after harvesting using a shovel at 40–50 cm depth. Each soil sample was a mix of 8–10 subsamples spread across the field to capture its variability. These analyses included the main physical parameters such as texture, colour, and pH, and chemical features (**Table 1**).

**Table 1:** Main physicochemical characteristics of conventional and organic soils in samples taken in 2024.

Parameters	Units	Conventional	Organic
Texture	%	Sand (78.74%)/Silt (12.42%)/Clay (8.84%)	Sand (54.22%)/Silt (24.50%)/Clay (21.28%)
pH (24.3°C)	–	7.5	7.9
Color	Munsell system	10yr 4/3 brown	10yr 6/3 pale brown
Conductivity	mS·cm^-1^	0.67	0.14
Nitrate	mg·kg^-1^	117	11
Nitric nitrogen	Nitrogen mg·kg^-1^	26.4	2.48
Assimilable phosphorous	mg·kg^-1^	71.6	47.5
Magnesium	meq·L^-1^	0.99	0.085
Assimilable potasium	mg·kg^-1^	137	135
Assimilable calcium	mg·kg^-1^	2910	1650
Assimilable magnesium	mg·kg^-1^	181	50
Soil organic matter (SOM)	%	2.26	3.23
Total organic carbon (TOC)	%	1.3	1.9
Cationic exchange capacity	meq·100g^-1^	14.9	8.75
Total nitrogen	%	0.136	0.212
Density	g·cc^-1^	1.48	1.35
Carbon/Nitrogen ratio	–	9.64	8.84

### 2.3 Rhizosphere DNA extraction and sequencing

DNA was extracted from 1 g of soil from each of the ten rhizosphere samples (five per farming system) using the NucleoSpin^®^ Soil DNA Extraction Kit (Macherey-Nagel, Germany). The composition and structure of the microbial rhizosphere communities were assessed through amplification and sequencing of the V3-V4 variable regions of the 16S rRNA gene for prokaryotes and the internal transcribed spacer (ITS) 2 region for fungi. The primers used for amplifying 16S rRNA were 341F (5’-CCTACGGGNGGCWGCAG-3’) and 785R (5’-GACTACHVGGGTATCTAATCC-3’), while ITS amplification was performed using primers ITS3 (5’-GCATCGATGAAGAACGCAGC-3’) and ITS4 (5’-TCCTCCGCTTATTGATATGC-3’). PCR amplification was conducted with 25 cycles. Negative controls were included to detect potential environmental contaminants, and a Mock Community (Zymo Research, California, USA) was used as a positive control. Library preparation and paired-end sequencing (2 × 300 bp) were performed on an Illumina MiSeq platform by Microomics Systems S.L.

### 2.4 Root RNA extraction and sequencing

Frozen common bean roots were mechanically homogenized using a TissueLyser II (QIAGEN, Aarhus, Denmark) equipped with a pre-chilled tube holder. The resulting powder was used for total RNA extraction with the NZY Total RNA Isolation Kit (NZYTech, Lisbon, Portugal), following the manufacturer’s instructions. RNA concentration and integrity were assessed using an Agilent 2100 Bioanalyzer and the Agilent RNA 6000 Nano Kit. RNA-seq libraries were prepared using the NEBNext Ultra II Directional RNA Library Prep Kit (New England Biolabs, Ipswich, MA, USA). mRNA was enriched by poly-A selection using the NEBNext Poly(A) mRNA Magnetic Isolation Module. The isolated mRNA was reverse transcribed into cDNA, and sequencing adapters were ligated to the fragments. Libraries were sequenced on an Illumina NovaSeq 6000 system with a paired-end 150 bp (PE150) flow cell by the company AllGenetics & Biology SL (A Coruña, Spain).

### 2.5 Bioinformatic analysis

Bioinformatic analyses were performed using two distinct pipelines: one for metabarcoding data and another for RNA-seq data.

#### 2.5.1 Metabarcoding

Raw demultiplexed forward and reverse reads were processed using QIIME2 (Bolyen et al., 2019). Quality control, including read trimming (phred score > 20), denoising, merging of paired-end reads, and chimera removal, was performed with DADA2 (Callahan et al., 2016), generating Amplicon Sequence Variants (ASVs). Multiple sequence alignment was conducted with MAFFT (Katoh and Standley, 2013). Taxonomic assignment was performed using the Bayesian Classifier (Wang et al., 2007) implemented in the SILVA database version 138 (Pruesse et al., 2007) using a pretrained classifier specific for the V3-V4 region of the 16SrRNA gene for prokaryotes (accessed on February 2025). Fungal ASVs were taxonomically assigned using the UNITE database version 8 (Kõljalg et al., 2005) with a 99% similarity reference dataset (accessed on February 2025).

To ensure comparability between samples and account for variability in sequencing depth, data were normalized using the rarefaction method. Rarefaction curves were generated with the *rarecurve* function from the “vegan” package in R (Oksanen et al., 2001) to evaluate the relationship between sequencing depth and observed diversity. Negative controls included in DNA extractions and library preparation yielded negligible read counts.

Alpha diversity, including richness, evenness, and the Shannon diversity index, were calculated from the average values of the replicates from each farming system. Richness was defined as the total number of ASVs per sample. Evenness was referred to uniformity of species abundances within a community, and was estimated using Pielou’s evenness index (Pielou, 1966). Differences in richness between farming systems were tested using a negative binomial model implemented in the “MASS” R package (Ripley and Venables, 2009) for richness, and the beta regression model in the “betareg” R package (Cribari-Neto and Zeileis, 2010) for evenness. Additionally, Pielou’s evenness was compared between rhizospheric samples using beta regression for both 16S and ITS data, which met the assumptions for this model, performed with the “stats” and “car” R packages (Fox et al., 2001). Additional tests (Shapiro–Wilk, t-test, and Wilcoxon rank-sum) were performed as exploratory analyses to evaluate distributional properties and robustness of the results. The Shannon diversity index (H’) was computed using the *diversity* function from the “vegan” package in R (Oksanen et al., 2001) with the formula H’ = -∑(pi * ln(pi)), where *pi* represents the proportion of reads assigned to ASV *i* in each sample. ASVs were used as proxies for species, and their relative abundances were derived from read counts.

Beta diversity was estimated based on phylogenetic distances between ASVs using the Jaccard similarity coefficient. Differences in beta diversity between farming systems were assessed using Permutational Multivariate Analysis of Variance (PERMANOVA) with the “vegan” R package (Oksanen et al., 2001). Principal Coordinates Analysis (PCoA) of the Jaccard distance matrix was used to visualize clustering patterns among samples. Pairwise differences between medians were evaluated using the Wilcoxon-Mann-Whitney test from the “stats” R package.

Differentially abundant microorganisms (DAMs) were obtained with the “metagenomeSeq” package v1.36.0 (Paulson et al., 2013) in R. Raw ASV count data were pre-processed to remove duplicate entries, and were then used to construct the phenotypic dataset for the MRexperiment object. To normalize for differences in library sizes, cumulative sum scaling (CSS) normalization was applied using the *cumNorm* function. DAMs were assessed using the *fitFeatureModel* function, which fits a zero-inflated log-normal model suited for sparse metagenomic datasets. ASVs with an adjusted *p*-value < 0.05 were considered significant DAMs.

Statistical analyses were performed using the R software v. 4.5.0 (R Core Team, 2024). In all cases, assumptions of normality (Shapiro-Wilk test, analyzed with “stats” R package) and homoscedasticity (Levene’s homogeneity of variance test, “car” R package) were studied before performing the corresponding statistical analyses. The significance threshold was set at *p* < 0.05.

Functional predictions of the prokaryotic community were performed with PICRUSt (Langille et al., 2013) using the file of ASVs observed as the input. Fungal ASVs were taxonomically parsed by ecological guild using the FUNGuild database (Nguyen et al., 2016) and its community-annotated database, processing the list of ASVs observed by species as input.

#### 2.5.2 RNA-seq

Raw reads were trimmed and filtered with Trimmomatic v.0.39 (Bolger et al., 2014) to remove Illumina adapter sequences and the first 15 bases from each read. Where average base quality was low (<25) reads were trimmed and any short reads (< 40 bp) were removed. The quality of the reads was then checked with FastQC v. 0.11.9 (http://www.bioinformatics.babraham.ac.uk/projects/fastqc/). Filtered reads were mapped to the *P. vulgaris* G19833 v. 2.0 genome (assembly accession: GCF_000499845.2; *Phaseolus vulgaris* v2.1, https://www.ncbi.nlm.nih.gov/datasets/genome/GCF_000499845.2/) using Minimap2 v2.17-r941 (Li, 2018). The resulting SAM files were converted to BAM format, sorted, and indexed using SAMtools v.1.10 (Li et al., 2009). Gene-level counts were obtained with HTSeq-count v. 1.99.2 using the *P. vulgaris* G19833 v. 2.0 gene annotation file in GTF format. The final output was a tab-delimited file containing raw counts for each gene. Genes with fewer than 10 total counts across all samples were removed prior to downstream analyses.

Normalization of RNA-seq count data and identification of root differentially expressed genes (DEGs) were performed with the “DESeq2” package (Love et al., 2014) in R, applying the likelihood ratio test. *P* values were adjusted using the Benjamini–Hochberg (BH) correction (Benjamini and Hochberg, 1995). Two filtering criteria were applied to identify DEGs: (1) a primary threshold based on the Benjamini–Hochberg FDR-adjusted p-value (≤ 0.05), and (2) a more restrictive criterion combining statistical significance (padj < 0.05) with |log_2_FC| ≥ 2.

DEGs were grouped and classified according to its Gene Ontology (GO) using the R package “clusterProfiler” (Yu et al., 2012), which performs functional enrichment analysis and visualization of biological themes among gene clusters. GO-related plots were generated with the “GOplot” R package (Walter et al., 2015). Additionally, the genomic locations of DEGs in common bean were visualized using the R package “karyoploteR*”* (Gel and Serra, 2017). The positional information of these genes was converted into *GRanges* objects and plotted onto the corresponding chromosomes. A karyotype plot was generated, displaying all chromosomes with base number annotations, providing a visual representation of their chromosomal distribution under OF conditions.

A targeted literature review was performed to compile genes previously associated with three functional categories: signaling, hormonal regulation, and development/nodulation. This gene set was cross-referenced with the DEGs from the RNA-seq analysis, and those with padj < 0.05 and |log_2_FC| ≥ 2 were considered for discussion.

### 2.6 Co-ocurrence matrix and correlations

To investigate significant associations between common bean root gene expression and microbial rhizospheric community, a cross-correlation analysis was performed between RNA-seq gene counts and microbial taxa abundances derived from metabarcoding data. Raw gene count tables and taxonomic abundance tables were first filtered using predefined lists of DEGs and DAMs (padj < 0.05, |log_2_FC| ≥ 2 in both cases). Variance Stabilizing Transformation (VST) was applied to the raw count matrix.

RNA-seq count data were normalized using the variance stabilizing transformation (VST) implemented in the “DESeq2” R package (Love et al., 2014) to ensure comparable variance across genes and enable robust visualization. Microbial abundance data were normalized using centered log-ratio (CLR) transformation with the “compositions” R package (van den Boogaart and Tolosana-Delgado, 2008). Pairwise correlations between normalized gene expression levels and microbial relative abundances were computed using Spearman’s rank correlation coefficient. Correlations and corresponding *p*-values were calculated using the *rcorr* function from the “Hmisc” R package (Harrell et al., 2025). Correlation heatmaps and annotated plots were generated with the “corrplot” (Wei et al., 2024) and “pheatmap” (Kolde, 2019) R packages.

## 3 Results

### 3.1 Field soil characteristics

Physicochemical soil analyses revealed some differences between the two soils collected (**Table 1**). Organic soil showed a higher pH, organic carbon, and organic matter content. In contrast, conventional soil showed higher conductivity, nitrate, assimilable magnesium and calcium.

### 3.2 Prokaryotic (16S) and fungal (ITS) sequencing alpha and beta diversity

The initial number of input reads ranged from 132, 834 to 1, 190, 189 for 16S sequencing and from 207, 703 to 866, 300 for ITS sequencing. After quality filtering, denoising, merging, and chimera removal, the number of observed ASVs ranged from 816 to 4, 277 for 16S and from 207 to 488 for ITS (**Supplementary Table 1**). The fungi-to-prokaryote ratio in rhizosphere samples was 0.11 under CF and 0.24 under OF, consistent with previously reported values for these soils (Suarez-Fernandez et al., 2025).

**Figure 1:**
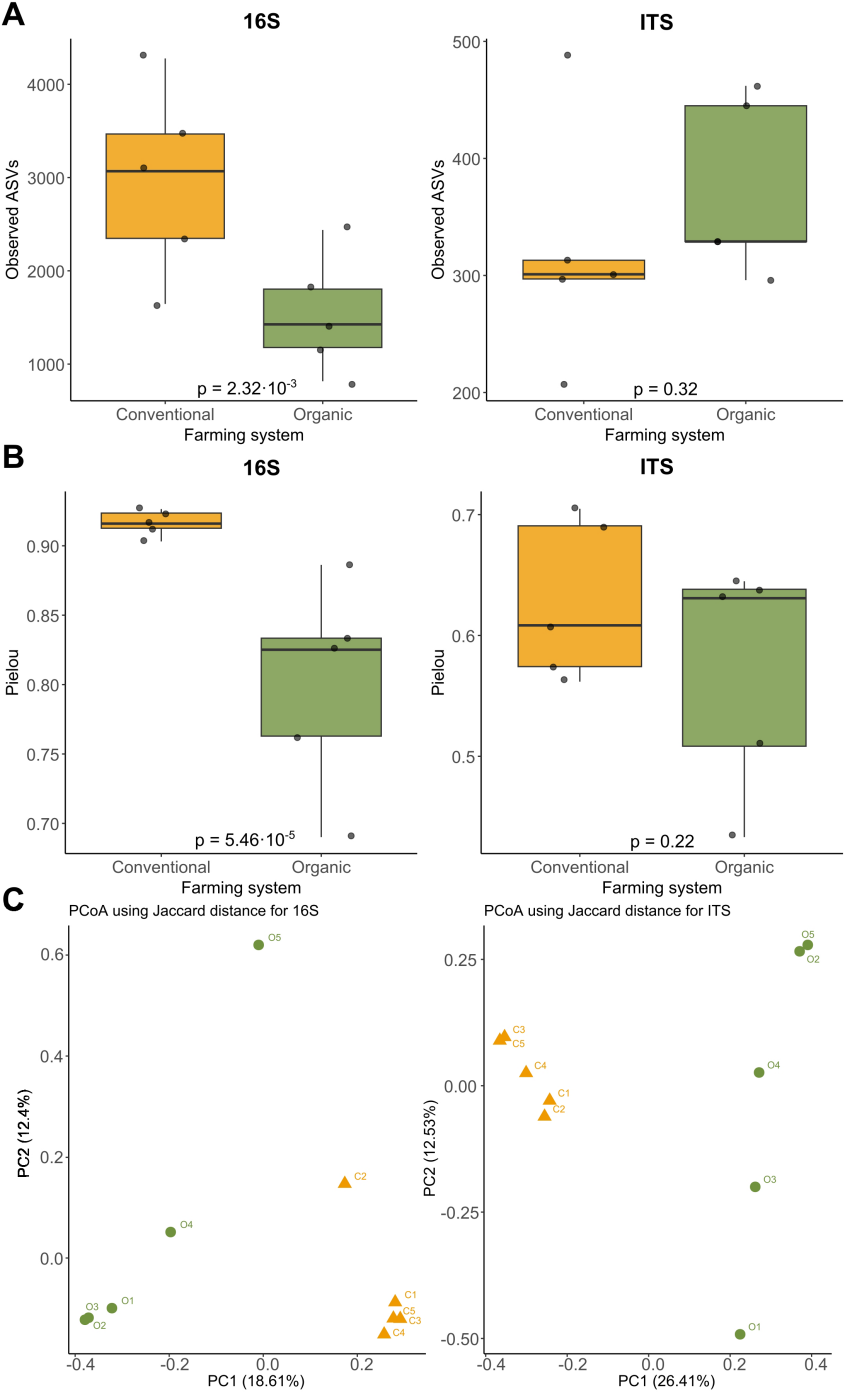
Alpha and beta diversities of common bean rhizospheric samples under organic (OF) and conventional farming system (CF). **(A)** Boxplots of alpha diversity (Observed Amplicon Sequence Variants (ASVs)) comparing organic and conventional samples, shown separately for 16S rRNA (left) and ITS (right). **(B)** Boxplots of community evenness based on Pielou’s index, for both 16S (left) and ITS (right), across farming systems, 16S rRNA (left) and ITS (right). **(C)** Principal Coordinate Analysis (PCoA) based on Jaccard dissimilarity distance for 16S (left) and ITS (right) communities. Green circles represent OF samples, and yellow triangles represent CF samples. Differences between farming systems were tested and are shown in **Supplementary Table 2**.

Rarefaction plots showed that the sequencing depth and subsampling size allowed (stabilization happened at 20000 sequencing depth) to capture the complete diversity present in prokaryotic (16S) and fungal (ITS) communities, reaching a plateau for the observed ASVs (**Supplementary Figure 3**). Alpha diversity measured as richness showed that prokaryotes were more abundant in the rhizosphere of CF than in OF (average 2, 961 ASVs observed vs. 1, 532.4, respectively). However, ITS observations showed the opposite trend (321.2 vs. 372.2; **Figure 1A**). Evenness, measured using Pielou’s index, is represented in **Figure 1B**. For 16S, significantly higher values were observed for CF samples than for OF samples. No significant differences were observed for ITS (**Supplementary Table 2**).

Estimation of Shannon diversity index (**Supplementary Table 2**; **Supplementary Figure 4**) in prokaryotic communities was 7.28 for CF and 5.81 for OF, revealing significant differences (Wilcoxon, p-value < 0.05). This index in fungal communities was 3.61 for CF and 3.38 for OF. For fungi, no significant differences were found in Shannon diversity index. Finally, beta diversity, which measures the variation in community composition between samples, was estimated using Jaccard’s similarity coefficients and visualized using PCoA (**Figure 1C**). The scatter plots showed high similarity between the samples according to the farming system. PCoA revealed two principal components from the 16S data, explaining 18.61% and 12.4% of the variation, respectively. For the ITS data, PCoA revealed two principal components explaining 26.41% and 12.53% of the variation, respectively. Statistical analysis of beta diversity revealed significant differences between farming systems for both prokaryotes and fungi (**Supplementary Table 2**).

### 3.3 Differences in microbial communities and functional analyses

The general analysis of relative taxonomic abundance according to farming system showed different distributions in both prokaryotes and fungal communities (**Supplementary Figure 4B**; **Supplementary Tables 3**, **4**). In prokaryotes, the proportion of the phylum Proteobacteria represented approximately 50% of the rhizosphere from OF, while in conventional represented approximately 15%. In conventional, phyla such as Bacteroidota, Acidobacteriota and Plactomycetota stood out. Regarding fungi, balanced proportions of taxa were present in both types of farming, but the main differences may be noted in phylum Rozellomycota (more prevalent in CF) and Basidiomycota (more abundant in OF, **Supplementary Figure 4C**).

Differential abundance analysis revealed distinct microbial profiles between farming systems (**Table 2**; **Supplementary Tables 5**, **6**). In organic rhizospheres, enriched prokaryotic taxa included genera such as *Allorhizobium-Neorhizobium-Pararhizobium-Rhizobium* and *Chitinophaga*, while dominant fungal genera included *Funneliformis*, *Conocybe*, *Minimedusa*, and *Metarhizium*. Conversely, CF soils showed higher relative abundance of bacterial genera like *Pirellula*, *Terrimonas*, and several *Planctomycetota*, alongside fungal taxa such as *Rhizophagus*, *Mortierella*, *Chaetomium*, and *Olpidium*. These results suggest that soil farming practices significantly shape the composition and potential functional capacity of both fungal and bacterial communities in the rhizosphere, potentially affecting plant–microbe interactions.

**Table 2:** Differential abundance analysis of fungal (ITS) and prokaryotic (16S) taxa in the rhizosphere of roots from conventional and organic systems was performed using the metagenomeSeq package.

Taxonomic annotation	log_2_FC	padj
Fungi > Basidiomycota > Agaricomycetes > Agaricales > Bolbitiaceae > Conocybe	6.48	5.21E-03
Bacteria > Proteobacteria > Alphaproteobacteria > Rhizobiales > Rhizobiaceae > Allorhizobium-Neorhizobium-Pararhizobium-Rhizobium	5.79	2.20E-11
Fungi > Glomeromycota > Glomeromycetes > Glomerales > Glomeraceae > Funneliformis > Funneliformis mosseae	5.44	0
Fungi > Basidiomycota 1	5.24	0
Fungi > Basidiomycota	4.99	2.80E-05
Bacteria > Bacteroidota > Bacteroidia > Chitinophagales > Chitinophagaceae > Chitinophaga > Chitinophaga sp.	4.91	1.67E-10
Fungi > Glomeromycota > Glomeromycetes > Glomerales > Glomeraceae > Funneliformis	4.84	0
Fungi > Basidiomycota > Agaricomycetes > Cantharellales > Cantharellales fam Incertae sedis > Minimedusa > Minimedusa polyspora	4.72	4.88E-11
Fungi > Basidiomycota > Agaricomycetes > Agaricales > Stephanosporaceae > unidentified > unidentified	4.71	2.09E-03
Fungi > Ascomycota > Sordariomycetes > Hypocreales > Clavicipitaceae > Metarhizium > Metarhizium marquandii	4.50	2.86E-13
Fungi > Glomeromycota > Glomeromycetes > Glomerales > Glomeraceae > Funneliformis > Funneliformis mosseae 2	4.42	0
Fungi > Glomeromycota > Glomeromycetes > Glomerales > Glomeraceae > Funneliformis > Funneliformis mosseae 4	4.32	0
Bacteria > Bacteroidota > Bacteroidia > Chitinophagales > Chitinophagaceae > unculture6	3.26	3.48E-06
Bacteria > Gemmatimonadota > Gemmatimonadetes > Gemmatimonadales > Gemmatimonadaceae > Gemmatimonas > uncultureGemmatimonadaceae	3.23	2.21E-03
Bacteria > Verrucomicrobiota > Verrucomicrobiae > Chthoniobacterales > Chthoniobacteraceae > Candidatus Udaeobacter > uncultureProsthecobacter 3	3.14	1.90E-01
Bacteria > Verrucomicrobiota > Verrucomicrobiae > Opitutales > Opitutaceae > Opitutus 3	3.08	2.21E-02
Bacteria > Bacteroidota > Bacteroidia > Chitinophagales > Chitinophagaceae > Flavisolibacter > Flavisolibacter ginsengisoli	2.99	8.10E-04
Bacteria > Actinobacteriota > Thermoleophilia > Gaiellales > Gaiellaceae > Gaiella 1	2.95	2.32E-02
Bacteria > Verrucomicrobiota > Verrucomicrobiae > Opitutales > Opitutaceae > Lacunisphaera 3	2.94	8.66E-06
Bacteria > Gemmatimonadota > AKAU4049 > AKAU4049 > AKAU4049 > AKAU4049 > unculturesoil	2.93	2.08E-02
Bacteria > Planctomycetota > Planctomycetes > Planctomycetales > uncultured > uncultured > unculturebacterium	-3.58	1.88E-02
Bacteria > Planctomycetota > Phycisphaerae > Tepidisphaerales > WD2101 soil group > WD2101 soil group > unculturebacterium	-3.59	1.88E-02
Bacteria > Planctomycetota > Phycisphaerae > Phycisphaerales > Phycisphaeraceae > AKYG587 > unculturebacterium 1	-3.59	1.88E-02
Bacteria > Myxococcota > bacteriap25 > bacteriap25 > bacteriap25 > bacteriap25 > unculturesoil 1	-3.63	0
Bacteria > Proteobacteria > Gammaproteobacteria > PLTA13 > PLTA13 > PLTA13 > unculturegamma	-3.68	1.88E-02
Bacteria > Bacteroidota > Bacteroidia > Cytophagales > Microscillaceae > Chryseolinea > uncultureBacteroidetes	-3.69	1.88E-02
Bacteria > Chloroflexi > Dehalococcoidia > S085 > S085 > S085 > unculturebacterium	-3.76	3.77E-03
Bacteria > Chloroflexi > Ktedonobacteria > C0119 > C0119 > C0119 > unculturesoil 1	-3.78	0
Bacteria > Bacteroidota > Bacteroidia > Chitinophagales > Chitinophagaceae > Terrimonas	-3.93	0
Bacteria > Planctomycetota > Planctomycetes > Pirellulales > Pirellulaceae > Pirellula > unculturebacterium	-4.33	0
Fungi > Ascomycota > Sordariomycetes > Sordariales > Chaetomiaceae > Chaetomium 1	-4.94	0
Fungi > Ascomycota > Leotiomycetes > Thelebolales > Pseudeurotiaceae > Pseudogymnoascus	-5.14	0
Fungi > Glomeromycota > Glomeromycetes > Glomerales > Glomeraceae > Rhizophagus	-5.19	0
Fungi > Glomeromycota > Glomeromycetes > Glomerales > Glomeraceae > Rhizophagus > unidentifie2	-5.23	0
Fungi > Olpidiomycota > Olpidiomycetes > Olpidiales > Olpidiaceae > Olpidium > Olpidium brassicae	-5.40	1.19E-03
Fungi > Ascomycota	-5.54	0
Fungi > Glomeromycota > Glomeromycetes > Glomerales > Glomeraceae > Rhizophagus > unidentified	-6.06	0
Fungi > Mortierellomycota > Mortierellomycetes > Mortierellales > Mortierellaceae > Mortierella	-6.15	0
Fungi > Glomeromycota > Glomeromycetes > Glomerales > Claroideoglomeraceae > Claroideoglomus	-6.23	0
Fungi > Rozellomycota > Rozellomycotina cls Incertae sedis > GS11 > unidentified > unidentified > unidentified	-8.77	0

The table shows the log_2_FC and Benjamini-Hochberg adjusted p-values (padj) for each taxon. Positive log_2_FC values indicate taxa that are more abundant in organic samples than in conventional samples, whereas negative log_2_FC values indicate taxa that are less abundant in organic samples. The full analysis is presented in **Supplementary Tables 6**, **7**.

Main bacterial functions were analyzed with PiCRUSt. In all, 4, 428 Clusters of Orthologous Genes (COGs) were associated to bacteria present in at least one of the samples, and 2, 433 of them showed significant differences between OF and CF communities (Wilcoxon, p-value < 0.05). The most remarkable functions in OF were transporters, kinases, and transferases, while in CF, the most remarkable functions were glycosyltransferases, dehydrogenases, among others (**Supplementary Table 7**; **Supplementary Figure 5A**).

Functional analysis was performed using FUNGuild to determine the main fungal lifestyle. A high abundance of symbiotrophic fungi was observed in the CF rhizosphere samples. In OF, a high proportion of saprotrophic fungi (probably due to the high presence of Basidiomycota) and fungi that can be either saprotrophic or symbionts, depending on their interaction with the plant, were found (**Supplementary Table 4**; **Supplementary Figure 5B**).

### 3.4 RNA-seq dataset, quality control and differential expression analysis

After filtering lowly expressed genes, a total of 22, 421 genes were retained for differential expression analysis out of the initial 32, 760 genes. Analysis of read count distributions revealed that, for most genes, variance across samples exceeded the mean, indicating overdispersion consistent with a negative binomial distribution and supporting the use of DESeq2 for differential expression analysis. PCA of the VST-normalized data revealed a strong separation of the samples by farming system along the first principal component (PC1), indicating that organic and conventional conditions induce distinct transcriptional profiles in common bean roots (**Supplementary Figure 6A**).

**Figure 2:**
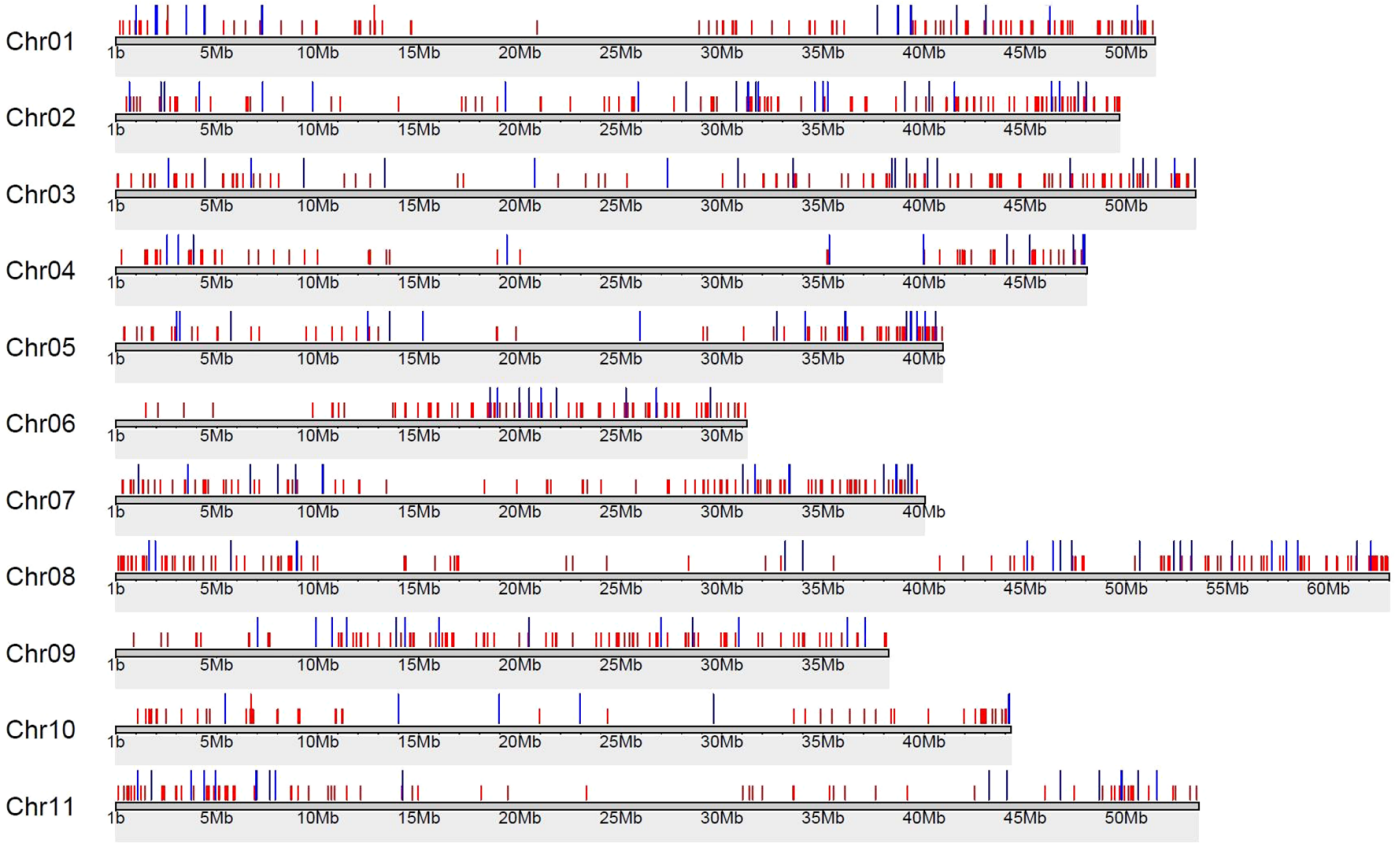
Distribution of DEGs (padj < 0.05; |log_2_FC| ≥ 2) on the *P. vulgaris* chromosomes: gray lines represent each chromosome, blue lines represent the genomic locations of downregulated genes, and red lines represent the genomic locations of upregulated genes.

A total of 5, 511 DEGs were identified, of which 2, 902 were upregulated and 2, 609 were downregulated in OF relative to CF (padj < 0.05; **Supplementary Table 8**). Chromosomal localization of DEGs (**Figure 2**) revealed a relatively stable distribution across the 11 bean chromosomes (~12–21%; **Supplementary Table 9**), with Chr09 showing the highest proportion (21.3%) and Chr10 the lowest (12.7%).

Considering the more restrictive criterion (|log_2_FC| ≥ 2, padj < 0.05), 1, 085 DEGs were identified showing an asymmetric distribution with a predominance of upregulated genes in OF samples (917 upregulated vs. 168 downregulated; **Supplementary Figure 6B**; **Supplementary Table 8**) suggesting an increased metabolic or regulatory activity.

### 3.5 Functional annotation and gene ontology enrichment

The top 20 of the 1, 085 DEGs (**Table 3**) included upregulated genes involved in secondary metabolism and stress responses, like cytochrome P450 83B1-like, cytochrome P450 81E8-like, serine acetyltransferase 4-like, and omega-6 fatty acid desaturase. Genes associated with protein modification and signaling, such as U-box domain-containing protein 35-like and WUSCHEL-related homeobox 1-like, also showed a strong change in expression. In contrast, the top-downregulated genes under OF included those associated with transcriptional regulation (e.g. MYB family transcription factor PHL5-like, WRKY transcription factor 41), nitrogen assimilation (nitrate reductase [NADH]_2_), and hormonal signaling (gibberellin 20-oxidase-like protein). Notably, redox-related genes such as monothiol glutaredoxin-S6-like, peroxidase 25-like, glutaredoxin-C1-like, and glutathione synthetase, are both up and downregulated (**Table 3**).

**Table 3:** The top 20 most upregulated and top 20 most downregulated DEGs in common bean roots under organic versus conventional systems.

Gene	log_2_FC	padj	Gene description
*LOC137818912*	12.03	3.91E-26	cytochrome P450 83B1-like
*LOC137818312*	10.61	2.29E-19	cytochrome P450 81E8-like
*LOC137805706*	10.42	2.62E-08	serine acetyltransferase 4-like
*LOC137827236*	10.15	1.20E-17	uncharacterized LOC137827236
*LOC137827760*	9.98	4.08E-15	omega-6 fatty acid desaturase, endoplasmic reticulum isozyme 1
*LOC137812772*	9.87	2.87E-13	U-box domain-containing protein 35-like
*LOC137830739*	9.38	1.81E-26	protein ZINC INDUCED FACILITATOR-LIKE 1-like
*LOC137836176*	9.31	1.52E-13	cycloartenol-C-24-methyltransferase-like
*LOC137827695*	9.07	1.61E-55	thiamine thiazole synthase, chloroplastic
*LOC137806270*	9.03	2.54E-12	glycosyltransferase BC10-like
*LOC137835422*	9.00	1.39E-10	shikimate O-hydroxycinnamoyltransferase-like
*LOC137814494*	8.87	2.45E-21	UDP-glycosyltransferase 73C2-like
*LOC137837953*	8.80	7.11E-11	WUSCHEL-related homeobox 1-like
*LOC137813850*	8.74	3.23E-10	photosynthetic NDH subunit of subcomplex B 2, chloroplastic
*LOC137812476*	8.72	1.93E-07	uncharacterized LOC137812476
*LOC137824618*	8.67	7.01E-16	glutaredoxin-C1-like
*LOC137819751*	8.66	1.47E-11	uncharacterized protein At1g28695-like
*LOC137814009*	8.41	3.87E-02	uncharacterized LOC137814009
*LOC137831721*	8.34	2.33E-27	glutathione synthetase, chloroplastic-like
*LOC137836535*	8.30	1.65E-08	thaumatin-like protein
*LOC137810153*	-3.33	2.91E-07	cytochrome P450 CYP82D47-like
*LOC137827147*	-3.34	6.21E-06	beta-glucosidase 24-like
*LOC137821185*	-3.38	1.59E-03	uncharacterized LOC137821185
*LOC137814148*	-3.39	6.91E-06	homeobox-leucine zipper protein HAT14-like
*LOC137806470*	-3.45	3.68E-02	proline-rich receptor-like protein kinase PERK3
*LOC137832952*	-3.46	5.98E-03	lysM domain receptor-like kinase 4
*LOC137805915*	-3.60	3.07E-03	peroxidase 25-like
*LOC137810535*	-3.61	2.75E-16	protein NRT1/PTR FAMILY 6.3-like
*LOC137806240*	-3.66	8.20E-07	small polypeptide DEVIL 13-like
*LOC137833164*	-3.66	4.39E-02	uncharacterized LOC137833164
*LOC137809370*	-3.75	3.67E-02	probable WRKY transcription factor 41
*LOC137831554*	-3.80	3.99E-02	transcription repressor OFP4-like
*LOC137829470*	-4.03	8.95E-04	uncharacterized LOC137829470
*LOC137825019*	-4.08	2.18E-10	gibberellin 20-oxidase-like protein
*LOC137816088*	-4.12	1.24E-03	monothiol glutaredoxin-S6-like
*LOC137826385*	-4.23	4.86E-51	nitrate reductase [NADH] 2
*LOC137812823*	-4.36	3.79E-03	protein DETOXIFICATION 51
*LOC137811690*	-4.72	9.61E-03	RING-H2 finger protein ATL74-like
*LOC137828797*	-5.34	1.60E-04	MYB family transcription factor PHL5-like
*LOC137814700*	-6.38	6.15E-06	monothiol glutaredoxin-S6-like

Genes are ranked by log_2_FC values obtained from DESeq2 analysis. Only genes with an adjusted p-value (padj) < 0.05 were considered.

A literature survey was conducted to identify root genes relevant to signaling, hormone-related responses, and development/nodulation. Among the DEGs, 74 matched these literature-derived candidates (**Supplementary Table 10**), and 22 of them met the significance thresholds (**Table 4**). These included genes involved in signaling, such as *ERECTA*, *IOS1*, *RGI5* and other *LRR-RLKs*, as well as the kinase *OST1*. Several hormone-related genes such as *EIN3*, *GA3OX1/GA4, IAA14, CKX5* and the ABA receptor *PYL6*, were also upregulated. Finally, genes associated with development and nodulation, such as *WAT1* (two *loci*), *ENOD93*, *NIN* and *NF-YC3*, were among the most highly expressed under OF, with log_2_FC values ranging from 2.61 to 7.24.

**Table 4:** DEGs of interest (log_2_FC ≥ 2 and padj < 0.05) identified from the curated literature-based gene set.

Gene	Symbol	Gene description	log_2_FC	padj
*LOC137836117*	EIN3	Ethylene insensitive 3 family protein	7.24	1.81E-06
*LOC137820337*	ERECTA LRR-RLK	LRR receptor-like serine/threonine-protein kinase ERECTA	5.83	4.13E-25
*LOC137835989*	GA3OX1, GA4	Gibberellin 3-oxidase 1	5.47	2.02E-03
*LOC137822825*	WAT1	WAT1-related protein At1g70260-like	5.27	4.92E-15
*LOC137808759*	OST1, P44, SNRK2-6, SNRK2.6, SRK2E	Serine/threonine-protein kinase SRK2E	5.03	1.03E-03
*LOC137835885*	IOS1 LRR-RLK	LRR receptor-like serine/threonine-protein kinase IOS1	4.91	1.09E-09
*LOC137814261*	IAA14, SLR	Indole-3-acetic acid inducible 14 (AUX28-like)	4.49	7.83E-07
*LOC137821397*	ENOD93	Early nodulin-93-like	4.44	3.07E-02
*LOC137821898*	CKX5	Cytokinin dehydrogenase 5	4.42	2.21E-05
*LOC137828722*	RGI5 LRR-RLk	LRR receptor-like serine/threonine-protein kinase RGI5	4.24	8.87E-07
*LOC137826037*	NF-YC3	Nuclear transcription factor Y subunit C-3-like	4.14	1.92E-03
*LOC137811803*	WAT1	WAT1-related protein At1g68170-like	3.59	2.54E-12
*LOC137822680*	NIN	Protein NLP2-like (PvNIN)	3.50	3.92E-02
*LOC137826364*	LRR-RLk	Leucine-rich repeat protein kinase family protein	3.39	3.27E-06
*LOC137832490*	PYL6, RCAR9	Abscisic acid receptor PYL12-like	3.28	3.70E-06
*LOC137825852*	LRR-RLk	Leucine-rich receptor-like protein kinase family protein	3.09	4.10E-03
*LOC137811670*	GSO1 LRR-RLk	LRR receptor-like serine/threonine-protein kinase GSO1	3.07	3.43E-04
*LOC137812752*	SWEET4-like	Bidirectional sugar transporter SWEET4-like	2.74	9.72E-08
*LOC137832988*	CRK25	Cysteine-rich receptor-like protein kinase 25 (CRK12)	2.66	7.46E-05
*LOC137811805*	WAT1	WAT1-related protein At1g25270-like	2.61	7.24E-08
*LOC137832396*	ENODL4	Early nodulin-like protein 4	2.56	1.42E-05
*LOC137826371*	DREB2C	Dehydration-responsive element-binding protein 2C-like	2.06	4.68E-04

The table lists genes associated with signaling, hormonal regulation, and development/nodulation that were significantly upregulated under OF. Additional information, including *Phvul* gene identifiers, chromosomal location, and supporting literature, is provided in **Supplementary Table 10**.

**Figure 3:**
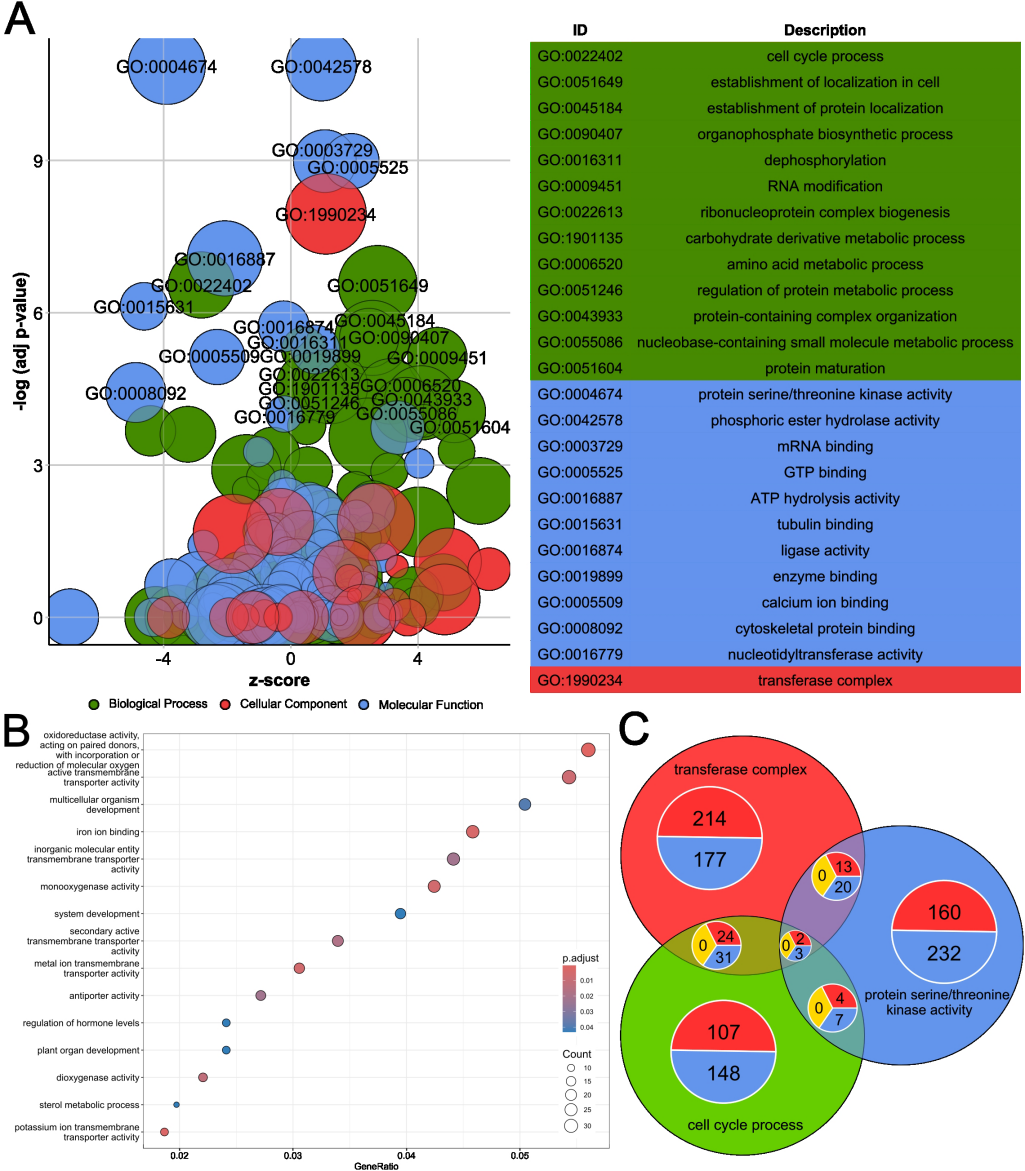
Gene Ontology enrichment and gene overlap across functional categories. **(A)** GO bubble plot generated using the GO plot package. Bubble size corresponds to the number of genes annotated to each term; color indicates the GO category: Biological Process (green), Molecular Function (blue), and Cellular Component (red). The x-axis shows the z-score (directionality of gene regulation), and the y-axis shows significance (-log10 padj). GO terms enriched among genes upregulated in the organic treatment (log_2_FC ≥ 2) and downregulated in the organic treatment (log_2_FC ≤ -2) are shown. **(B)** Dotplot of significantly enriched GO terms (padj < 0.05) in upregulated genes under the organic treatment. Dot size reflects the gene count per term and color indicates the adjusted p-value. **(C)** Venn diagram showing the overlap of genes among three representative GO terms, one from each ontology. The color of each circle corresponds to the GO plot category. Shared and unique genes, as well as their regulation direction (based on log_2_FC), are displayed (red up, blue down, yellow different directions).

GO enrichment analysis across was performed in the 5, 511 DEGs considering Biological Process (BP), Molecular Function (MF), and Cellular Component (CC) categories. **Figure 3A** displays a GO bubble plot showing significantly enriched GO terms (padj < 0.05). Terms such as “establishment of localization in cell”, “protein maturation”, “RNA modification”, “mRNA minding”, “GTP binding”, or “transferase complex” were enriched among genes upregulated in OF, while “cell cycle process” and “protein serine/threonine kinase activity”, “tubulin binding”, “cytoskeletal protein binding”, “calcium ion binding”, or “ATP hydrolysis activity” were associated with downregulated genes (**Supplementary Table 11**). Complementarily, we investigated the top enriched GO terms (BP and MF) among genes significantly upregulated in OF, showing consistent enrichment in signaling, transport and developmental processes (**Figure 3B**). To further dissect gene-level overlap between selected key terms, we selected the most significant GO term in each GO category: “cell cycle process” for BP, “protein serine/threonine kinase activity” for MF and “transferase complex” for CC. The intersection of the three processes is shared by 5 genes (**Figure 3C**; **Supplementary Table 11**): 2 upregulated (*LOC137831531* - cell division control protein 2 homolog, and *LOC137826316* - cell division control protein 2 homolog C-like) and 3 downregulated (*LOC137816276* - cyclin-dependent kinase B2-2, and *LOC137811786* and *LOC137831983* - cell division control protein 2 homologues).

### 3.6 Co-occurrence matrix

**Figure 4:**
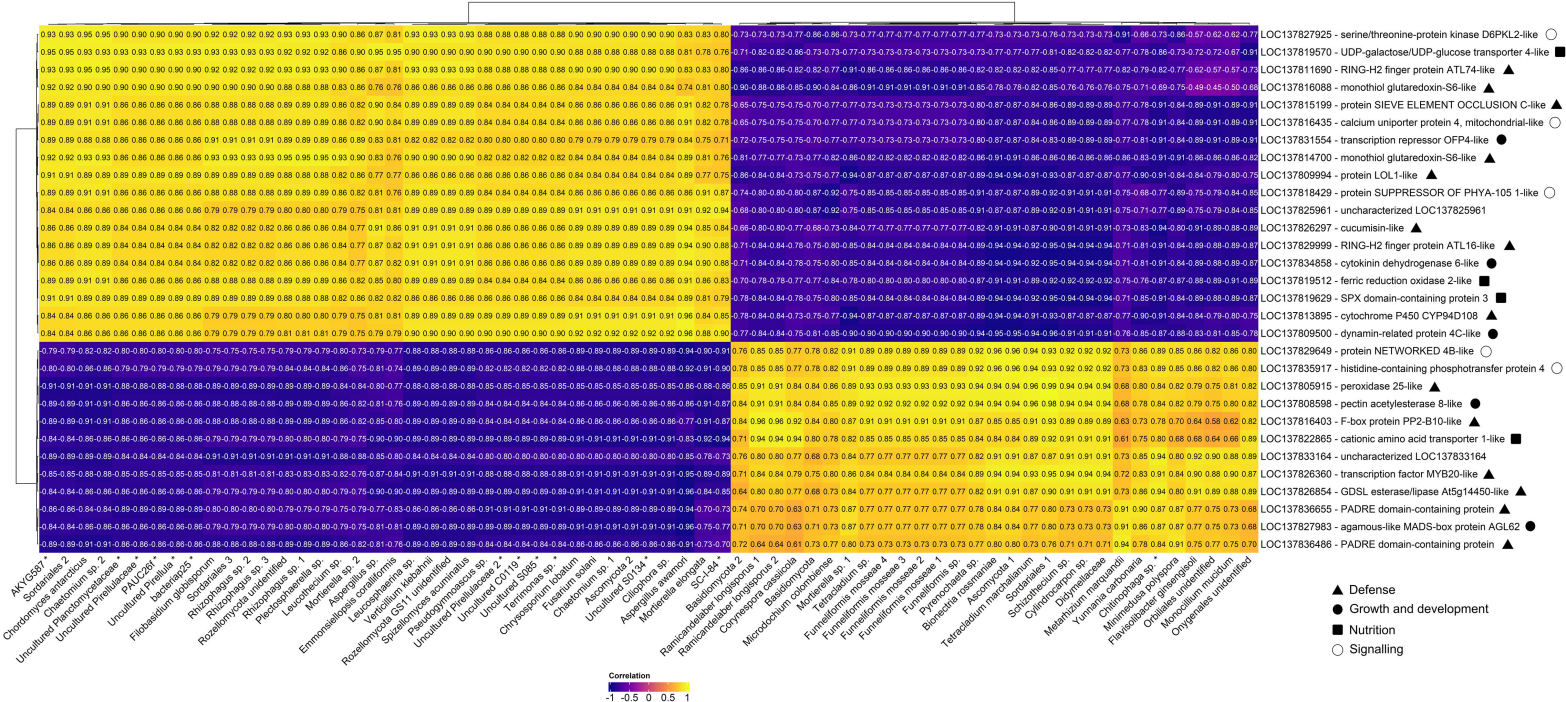
Heatmap of host–microbiome interactions based on Spearman correlations between the top 30 differentially expressed root genes (DEGs) and their most strongly associated microbial taxa (|R| ≈ 1). Only DEGs and differentially abundant microbes (DAMs) with |log_2_FC| ≥ 2 and padj < 0.05 were included. Prokaryotic taxa are indicated by asterisks, and putative gene functions are represented by triangles, circles or squares according to the legend on the bottom right. Purple boxes indicate negative correlations and yellow boxes indicate positive correlations.

The 30 common bean root genes with the highest differential expression values and the ASVs with a higher significant correlation with those genes were selected. Hierarchical clustering of the correlation matrix revealed patterns of association between rhizosphere microorganisms and root gene expression profiles (**Figure 4**), with correlation coefficients ranging from moderate to very strong (approximately |r| = 0.6–0.95).

Two major clusters with contrasting correlation patterns were identified. Cluster A (left part in **Figure 4**), predominantly positively correlated with CF, grouped mixed bacterial taxa (e.g., uncultured *Terrimonas*, *Pirellula*) and pathogenic (*Fusarium solani*) or saprotrophic fungi (*Mortierella*). Within this module, several plant genes displayed consistently strong positive correlations with these microbial taxa. Representative genes included those involved in stress protection and redox homeostasis such as monothiol glutaredoxin S6-like (*LOC137816088*), cytochrome P450 family proteins (*LOC137813895*), and ferric reduction oxidase 2-like (*LOC137819512*), as well as regulatory components such as serine/threonine protein kinase (*LOC137827925*) Genes associated with tissue protection and repair (sieve element occlusion C-like proteins – *LOC137815199*), and growth modulation mechanisms (cytokinin dehydrogenase 6-like – *LOC13783485*, and transcription repressor OFP4-like – *LOC137831554*) were also strongly represented.

In contrast, Cluster B (right part in **Figure 4**) was mainly associated with OF and enriched in beneficial fungal groups, including taxa involved in biocontrol (*Metarhizium*, *Bionectria*) and arbuscular mycorrhizal fungi (*Funneliformis*) linked to nutrient absorption. This cluster showed predominantly strong positive correlations among beneficial microbial taxa and plant genes involved in active defense mechanisms, growth-related processes, and nutrient uptake efficiency. Representative genes included peroxidase 25-like (*LOC137805915*), GDSL esterase/lipase At5g14450-like (*LOC137826854*), and transcription factor MYB20-like (*LOC137826360*), among others. These associations reflect a balance between plant immunity, development, and resource acquisition in soils with OF.

## 4 Discussion

This study investigated the variation in the rhizosphere microbiome of a common bean genotype grown under two farming systems: organic and conventional. In parallel, root gene expression was analyzed to characterize the transcriptomic response of common bean to these contrasting soil conditions. Integrating both datasets provide insights into how farming practices influence the rhizosphere microbiome and the plant’s molecular response. Both field trials (organic and conventional) were conducted under identical climatic conditions and in soils with well-known history and characteristics, suggesting that the observed differences were primarily driven by the historical farming system.

Our results revealed higher bacterial diversity in the rhizosphere under CF, whereas fungal diversity was greater under OF. These patterns are consistent with previous analyses of the microbiome of the same soils (Suarez-Fernandez et al., 2025) and might be related to the use of pesticides in CF. These findings suggest a potential link between bulk soil and rhizosphere microbial diversity. In addition, the rhizospheric fungi-to-prokaryote ratio doubled under OF, indicating a more favourable microbial structure compared to CF. Clear shifts in differentially abundant taxa were observed when comparing both studies (bulk soil vs. rhizosphere communities). In the soil analysis, CF was characterized by a higher abundance of *Bacillus*, *Sphingomonas*, and members of the order *Azospirillales*, while OF soils were enriched in archaeal taxa such as *Nitrososphaeria*. In contrast, the rhizosphere study revealed a distinct microbial profile, likely reflecting the selective influence of the root environment (Saleem et al., 2018). In CF soils, *Pirellula* and *Terrimonas* were prominent, whereas OF favoured *Chitinophaga* and the *Allorhizobium–Neorhizobium–Pararhizobium–Rhizobium* complex, consistent with recruitment of nitrogen-fixing and chitin-degrading bacteria (Dubey et al., 2022). This rhizobia recruitment in OF is highly relevant for their role in enhancing soul fertility and yield (Shah et al., 2024). For fungi, a similar shift was observed. In bulk soil, *Rhizophagus* was more abundant under OF, whereas CF soils were enriched in *Botryotrichum*, *Ramicandelaber*, *Mortierella*, *Gamsia*, and *Cladosporium* (Suarez-Fernandez et al., 2025). In the rhizosphere, OF favored the presence of Basidiomycota and *Funneliformis*, while CF was characterized by *Rozellomycota*, *Claroideoglomus*, *Mortierella*, *Rhizophagus*, *Ascomycota*, and *Olpidium*. These differences suggest that the transition from soil to rhizosphere amplifies plant-driven microbial recruitment (Yang et al., 2024), likely reflecting functional adaptations to each farming system. Nevertheless, this contrasts with Park et al. (2023), who found no statistically significant differences in common bean rhizosphere diversity under organic and conventional systems. The use of populations instead of a single bean genotype, as in the present study, may have masked genotype–specific microbiome interactions.

Phenotypic differences in roots were observed between farming systems. Bean roots grown under OF had visible lateral branches and more abundant nodules than in CF. These traits suggest an enhanced capacity for nutrient uptake and symbiotic interactions, possibly driven by the more diverse and functionally active microbiome associated with OF (Fortu et al., 2025). Root architecture is highly plastic and responds dynamically to soil properties such as texture, nutrient availability, and microbial activity (Yetgin, 2024). In common bean, multiple QTL associated with root traits—including length, diameter, and branching—distributed across 11 chromosomes, highlighting the complex genetic control underlying these phenotypes (Singh et al., 2019; Strock et al., 2019; Ambachew and Blair, 2021). Root architecture also responds to soil chemical and biological conditions, including microbiome composition. Soil microorganisms can interact chemically with plants via root-derived signals, influencing hormonal pathways and shaping root development (Dini‐Andreote et al., 2025).

Transcriptomic analysis revealed 1, 085 DEGs in common bean roots under OF, many related to root growth, nodulation, hormonal regulation, and plant–microbe interactions. Overall, the genes upregulated under OF revealed a coordinated physiological adjustment that strengthens root development, defense capacity, and symbiotic performance. Activation of regulators of root architecture (e.g., *GA3OX1*, *IAA14, WAT1, RGI5*, *IOS1*) in OF suggests more resources in root exploration, vascular remodeling, and lateral root formation, likely to compensate for lower nutrient availability in the soil (Fukaki et al., 2002; Yeh et al., 2016; Sun et al., 2021; Sánchez-Correa et al., 2022). In parallel, increased expression of stress- and pathogen-responsive components such as *EIN3*, *OST1/SRK2E*, *PYL6*, and several LRR receptor-like kinases (*LRR-RLKs*) points to a more active defensive state, potentially reflecting greater microbial complexity or environmental variability in organic systems (Goff and Ramonell, 2007; Kulik et al., 2011; Miao et al., 2018; Akbulut et al., 2023). Notably, the upregulation of nodulation-related genes (*NIN, ENOD93, NF-YC3*) further indicates an increased reliance on biological nitrogen acquisition and a tighter coordination between root development and symbiotic signaling (Zanetti et al., 2011; Liu and Bisseling, 2020; Lee et al., 2024). Together, these responses underscore the capacity of common bean to finely tune growth, defense, and symbiotic pathways, enabling a more efficient exploitation of soil resources and microbial interactions under OF.

Apart from those already mentioned, several nodulation-related genes (*nodulin30*, *ENOD8*) were also upregulated, supporting the idea that OF promotes active nodulation and symbiotic nitrogen fixation as a compensatory mechanism for reduced inorganic nutrient availability (Barbieri et al., 2023). Moreover, DEGs associated with the synthesis of root exudate metabolites—such as fatty acids, indolic compounds, and organic acids—were identified. These act as key substrates or signaling molecules for microbial recruitment (Farmer, 1994; Venturi and Keel, 2016). Transcriptomic signatures suggest that plants under OF may actively modulate their exudation patterns to attract beneficial microorganisms, thereby enhancing nutrient acquisition, growth, and stress tolerance.

This study reveals the impact of the crop system on the rhizosphere microbial community and the differential response of root genes of a genotype to these two environments. The observed variation and specific interaction should also be explored in other bean genotypes and environments. The identification of key genes involved in root development and plant–microbe interactions has important implications for breeding programs aimed at developing more resilient and efficient cultivars (Pantigoso et al., 2022; Araujo et al., 2025). Genes associated with microbial recruitment could be particularly valuable for selecting genotypes better adapted to low-input, stress-prone environments such as OF systems, where plants rely more heavily on beneficial microbial partnerships. Likewise, genes controlling root offer potential targets for improving architecture and nutrient uptake efficiency root system performance under both conventional and organic conditions. Nevertheless, the large number of genes contributing to these complex traits suggests that breeding directly under organic conditions may represent the most effective strategy to capture the optimal gene–microbiome combinations that underpin plant performance in such environments.

In conclusion, these findings highlight the importance of considering plant–soil–microbiome interactions as an integrated system when designing sustainable agricultural strategies and future crop improvement programs.

## Data Availability

Metabarcoding data of this study are open and available in the ENA at EMBL-EBI (www.ebi.ac.uk) under the accession numbers PRJEB98592 (ITS) and PRJEB98591 (16S). RNA-seq data are open and available in NCBI (www.ncbi.nlm.nih.gov) under the accession number PRJNA1337613.
