# Supplementary material for: Farming system shapes rhizosphere microbiota and root gene expression in common bean
Source: Front Plant Sci. 2026 Mar 26;17:1749874. doi: 10.3389/fpls.2026.1749874 (PMC13062228; doi:10.3389/fpls.2026.1749874)
Supplement: Supplementary file 1 [file DataSheet1.pdf]

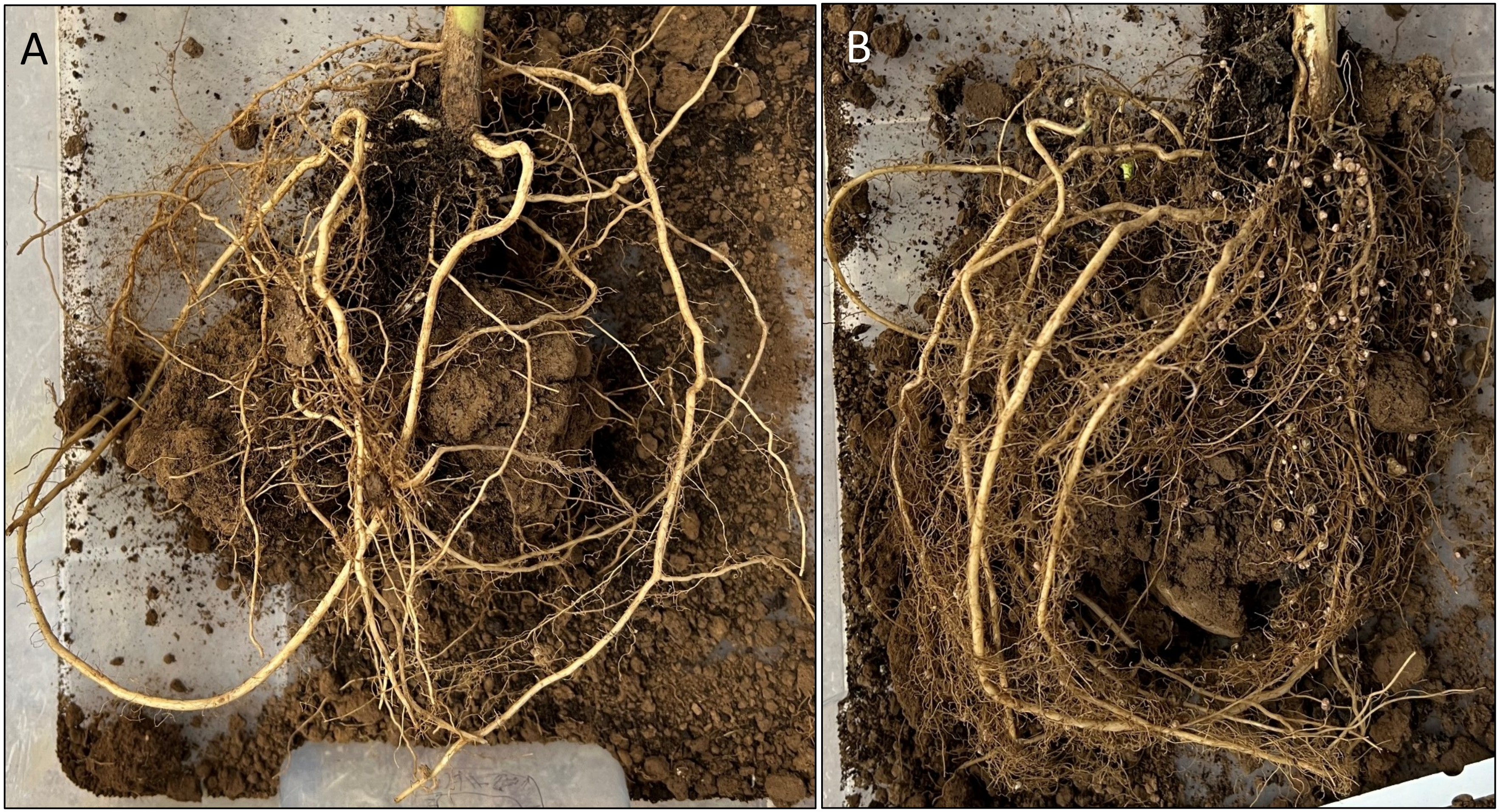

Supplementary Figure 1. Roots from the cultivar A25 obtained under A) conventional and B) organic farming systems. Roots from the organic treatment exhibited a higher abundance of nodules and secondary roots than those grown under conventional treatment.

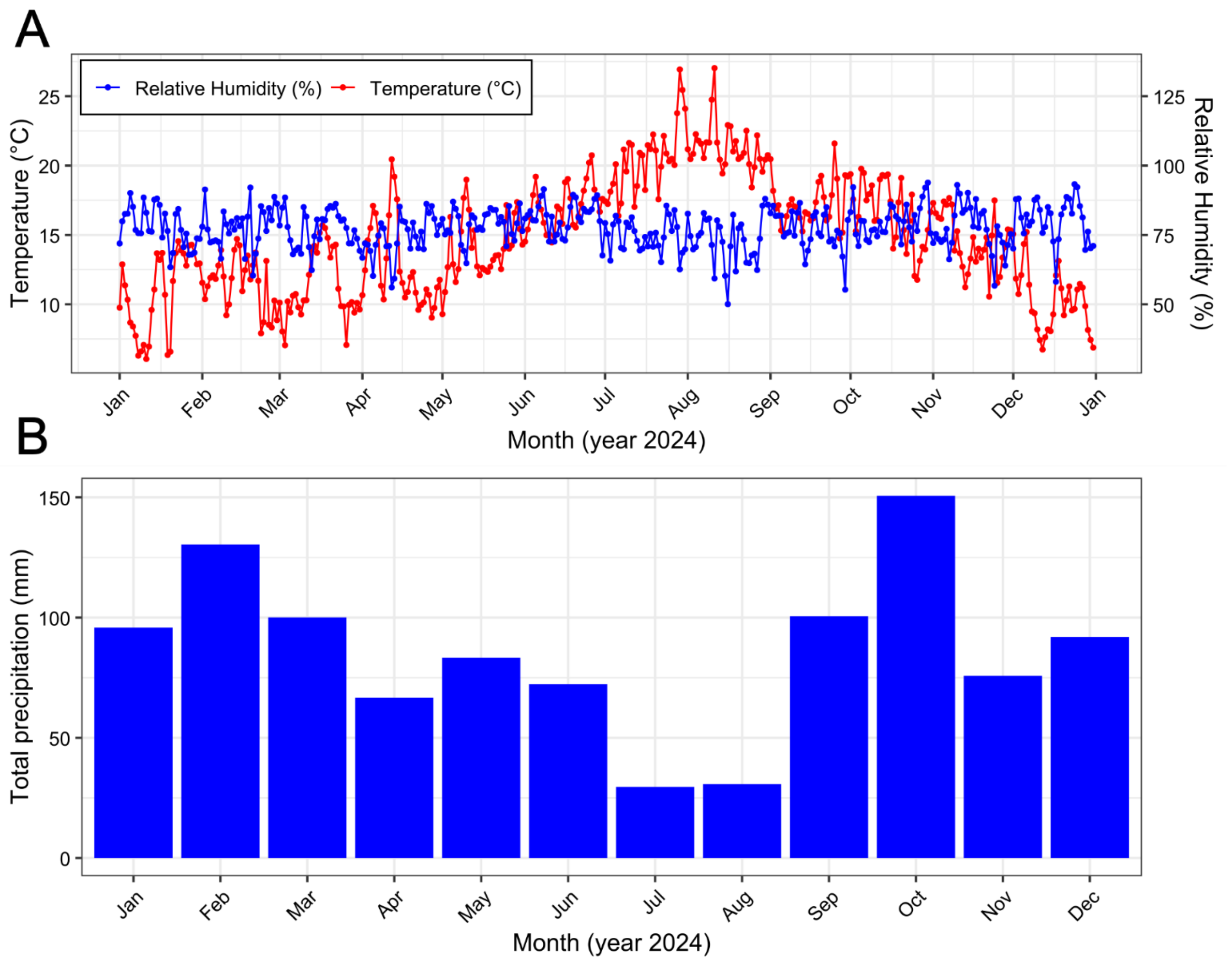

Supplementary Figure 2. Climatic data from January 1 to December 31, 2024. A) Daily mean temperature (left Y-axis, red line), and mean relative humidity (right Y-axis, blue line). B) Accumulated precipitation (mm) per month. Data represent local environmental conditions during the experimental period. Roots were collected by July 15th, 2024. Source: https://power.larc.nasa.gov/data-access-viewer/

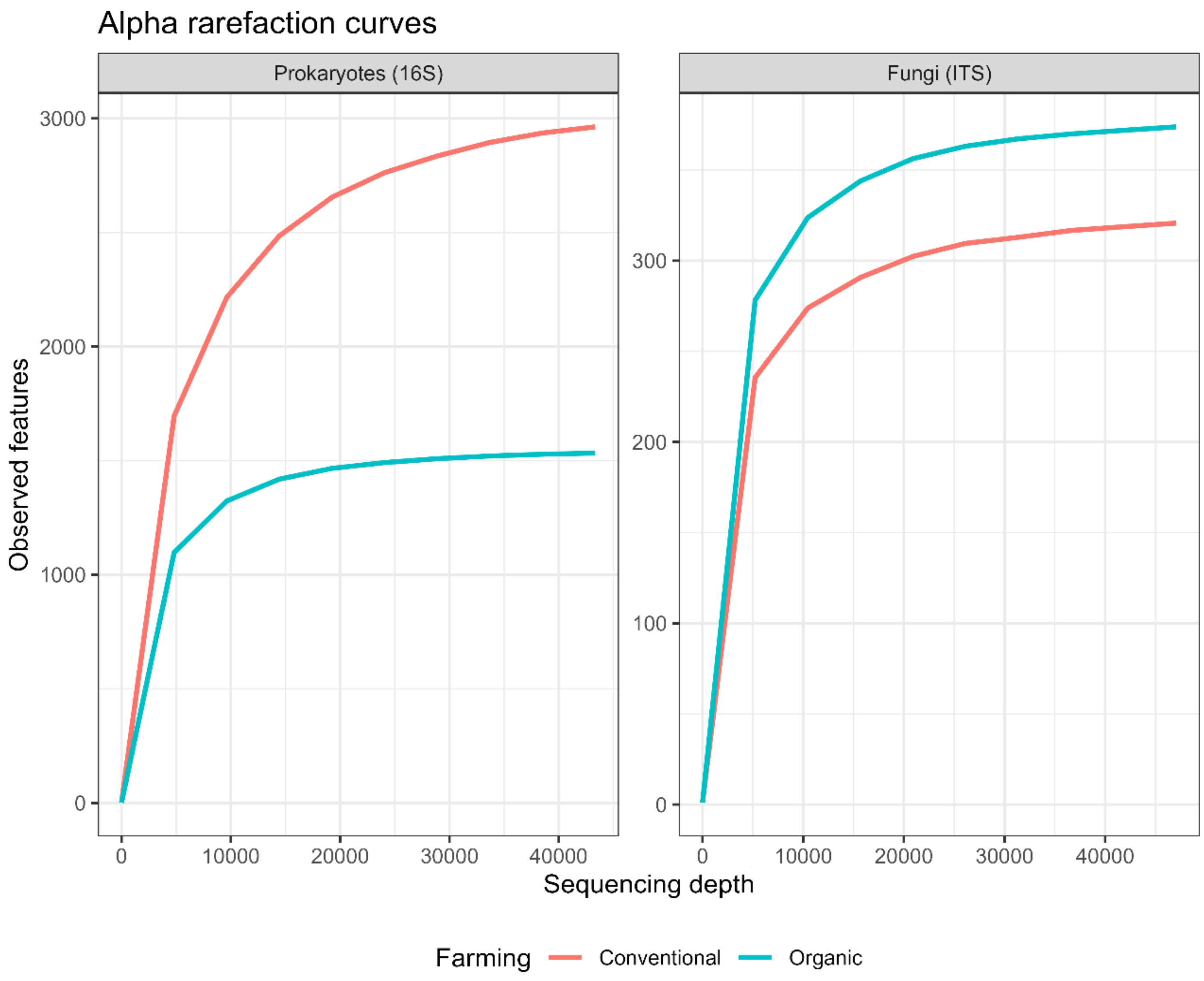

Supplementary Figure 3. Rarefaction plots. Left: Prokaryotic (16S) samples were subsampled up to 43,340 reads to even sample size and make quantitative comparisons. Right: Fungal (ITS) samples were subsampled up to 47,060 reads.

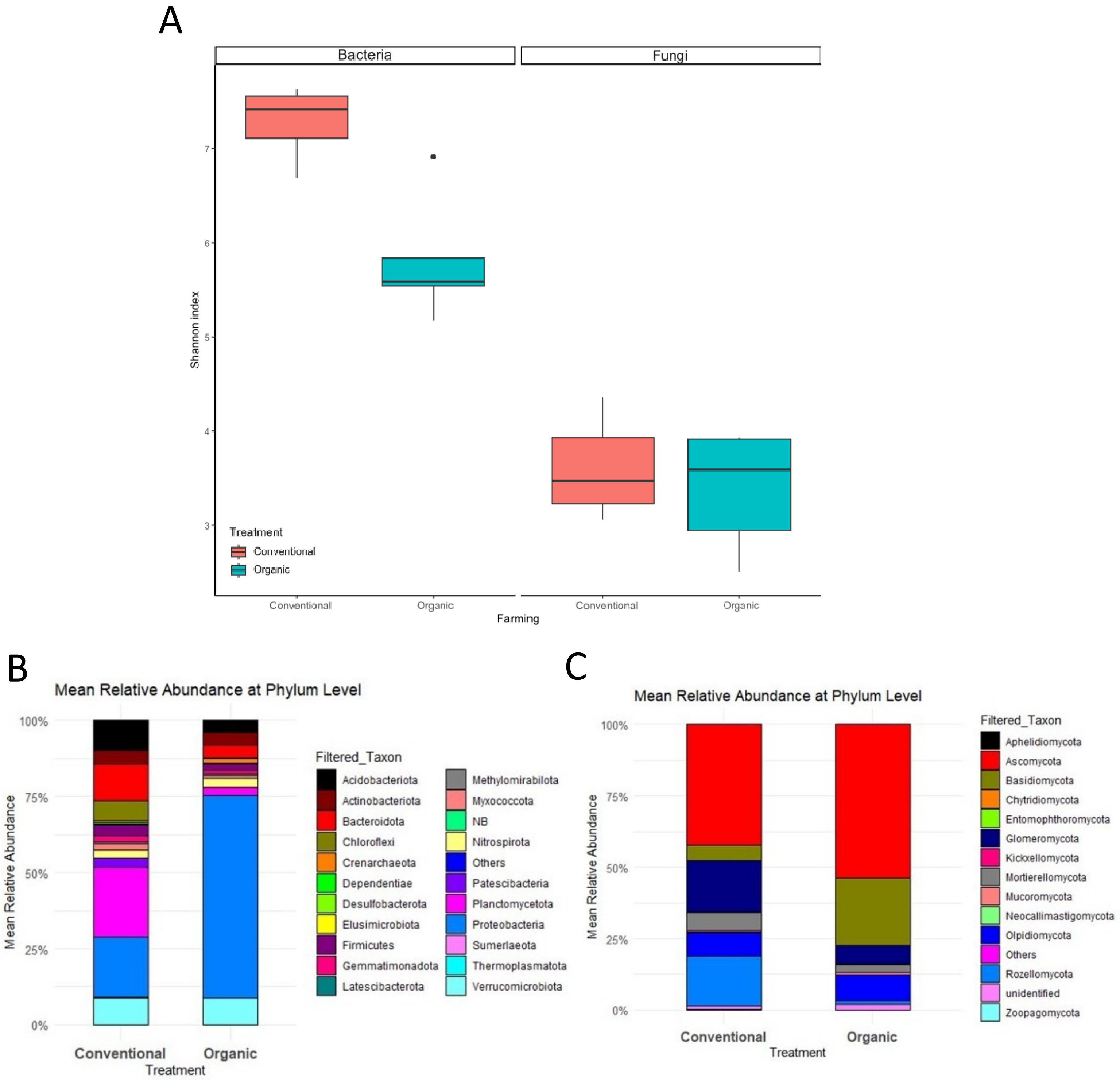

Supplementary Figure 4. Additional diversity metrics for rhizosphere microbial communities. A) Shannon diversity index of bacterial (16S) and fungal (ITS) communities under organic and conventional farming systems. B) Mean relative abundance of bacterial phyla (16S). C) Mean relative abundance of fungal phyla (ITS).

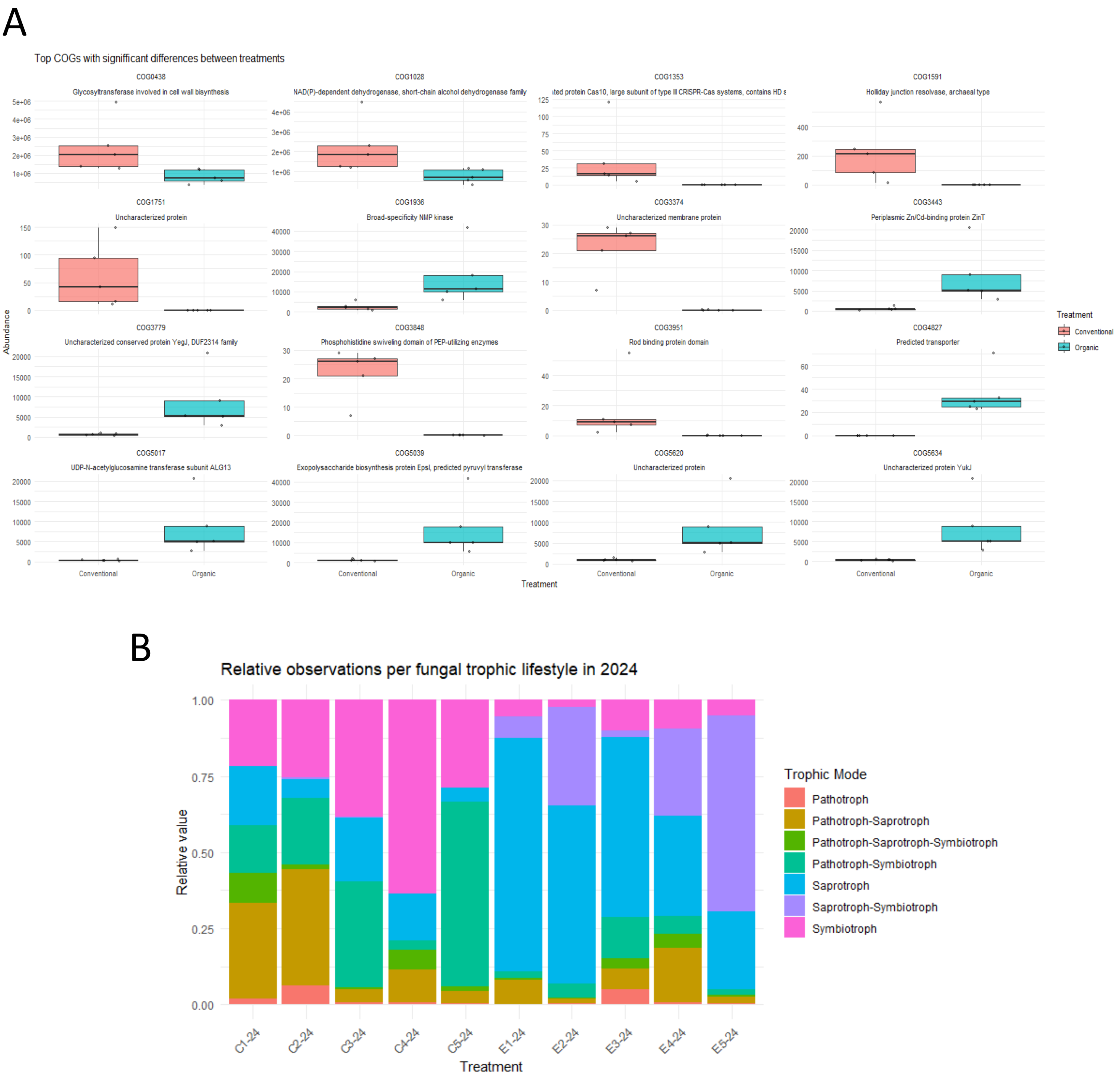

Supplementary Figure 5. Functional analyses of A) prokaryotes, analyzed with PiCRUSt; and B) fungi, analyzed with FUNGuild.

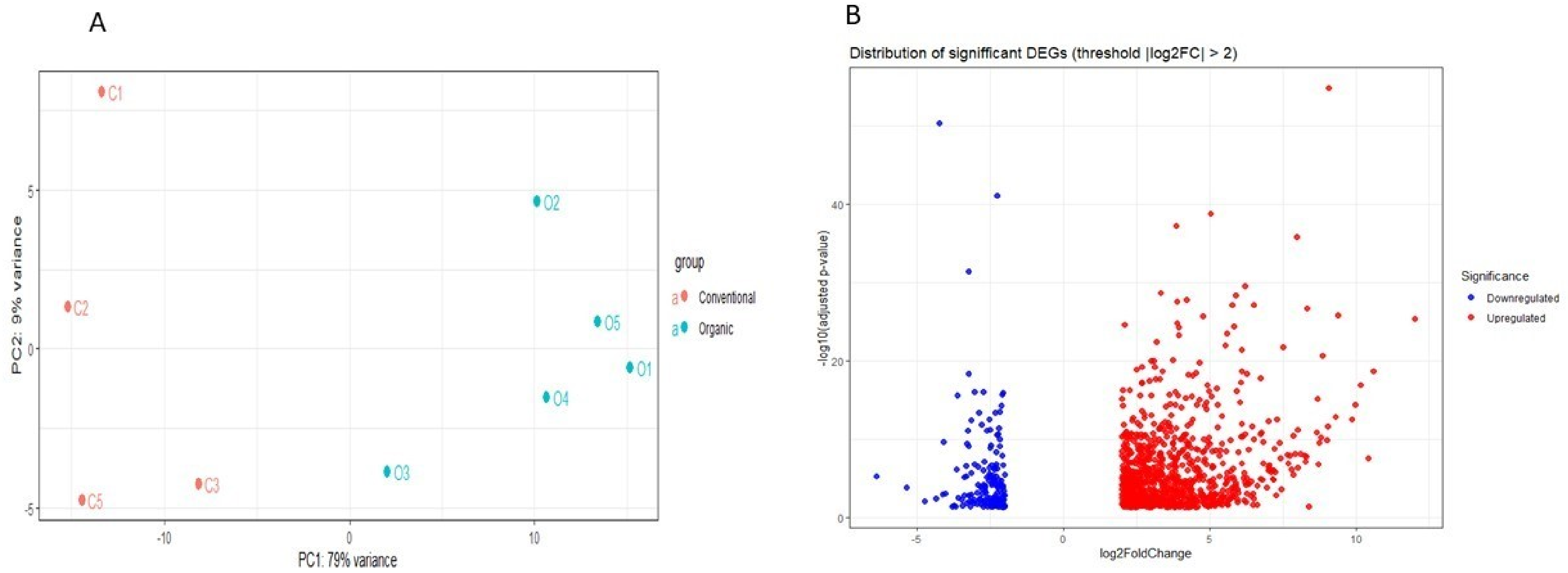

Supplementary Figure 6. A) Biplot with the results of Principal Component Analysis (PCA) from Variance stabilizing transformation (VST) -normalized data. *B) Volcano plot of differentially expressed genes with |*log_2*FoldChange| > 2.*
